# Institutionalization of Health System to Combat the COVID-19 Pandemic in Iran

**DOI:** 10.3389/ijph.2023.1606197

**Published:** 2023-07-18

**Authors:** Hajar Haghighi, Amirhossein Takian, Azam Raoofi

**Affiliations:** ^1^ Department of Health Management, Policy and Economics, School of Public Health, Tehran University of Medical Sciences, Tehran, Iran; ^2^ Department of Global Health and Public Policy, School of Public Health, Tehran University of Medical Sciences, Tehran, Iran; ^3^ Health Equity research center (HERC), Tehran University of Medical Sciences, Tehran, Iran

**Keywords:** COVID-19, pandemic, Iran, health system, institutionalization

## Introduction

The COVID-19 crisis and the inevitable future pandemics are expected to affect not only the health of nations, but also their socio-economic characteristics. Pandemics are largely addressed with a biomedical approach. Nonetheless, the COVID-19 pandemic has reminded all health authorities to move from this perspective to a more sustainable institutional arrangement, especially in the global context of many uncertainties [1]. It is essential to ensure a functional institutional arrangement in the context of health systems through the generation of policy unit members, which may in turn conduct predominant functions in a routinized manner that will become standard practice [2].

**FIGURE 1:**
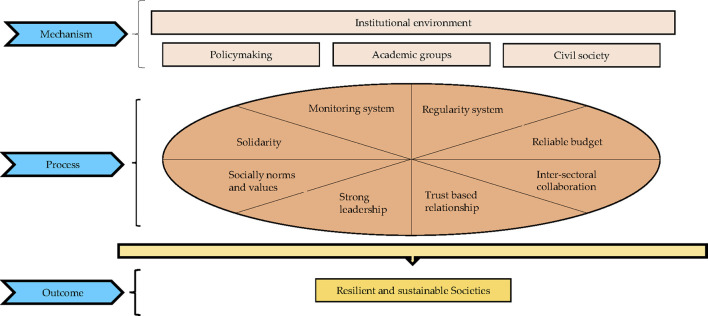
The underpinnings of institutional arrangement (The figure is originally created by authors) (Iran, 2020).

Institutionalization is an organized system of society relations, which shapes political officials’ behavior [2]. Such an environment is about stakeholders who should take a step back from their predetermined frameworks and make decisions for the population’s health and wellbeing. This perspective requires more solidarity, reinforcing socially embedded norms and values, a reliable budget, a sophisticated monitoring system, enacting regulations, inter-sectoral collaboration, and strong leadership (Figure 1) [1]. Institutionalization facilitates the involvement of ideas into organizational structures, which might in turn result in automating actions in any situation, i.e., emergencies [3]. Such an approach is expected to promote countries’ surveillance capacity in response to international public health emergencies, as also endorsed by the International Health Regulations (IHR), as well as the pandemic treaty [4].

The story of various countries’ experiences in combating COVID-19 reveals the boosting effect of sustainable institutional arrangement on their outcome [5]. Some nations understood the seriousness of the pandemic rapidly and showed a relatively better performance to mitigate and control the crisis compared to others. It is noteworthy that these organized systems also focus on the management and coordination of government operations in emergency situations [6].

According to the institutional approach, individual-organizational capacity development and strong inter-sectoral collaboration are both the essential pre-requisites to combat the crisis [7]. To reach this goal, multi-sectoral committees were established to manage the pandemic and hinder differential detrimental decisions. These committees have resulted in united command and integrated decisions [6]. Hence, addressing institutional arrangement in all government sectors, i.e., health, is crucial to minimize the adverse impact of uncertainties on the sustainability of all societies.

## Institutionalization, Health System and COVID-19 in Iran

Since the official announcement of two deaths due to COVID-19 on 19 February 2020, Iran has remained among the top 15 countries regarding the total confirmed cases; and the first country in the Eastern Mediterranean Region in terms of mortality and morbidity. On 3 January 2022, the Iranian Ministry of Health and Medical Education (MoHME) declared 194 confirmed cases of the new COVID-19 variant, which spread rapidly in a dozen Iranian provinces [8]. Enjoying a comprehensive Primary Healthcare (PHC) network, greater resiliency was expected from Iran’s health system to mitigate morbidity and mortality associated with the COVID-19 pandemic [9]. However, the double burden of unilateral political sanctions [10], and delays in making some critical decisions after declaring the “Global Emergency,” led to the rapid spread of the virus around the country [11].

The National COVID-19 Committee (NCC) was established to respond to this global emergency. The NCC attempted inter-sectoral collaboration and the involvement of various relevant actors [12]. Despite its efforts, the NCC faced challenges in fostering a meaningful inter-sectoral collaboration and integrated decision-making [11]. The inadequate coordination among policymakers, as well as dysfunctional channels of communication among various government sectors, led to incoherent measures and remarkable inconsistencies in relation to COVID-19 decision-making [10]. Consequently, the country faced pandemic waves one after another, mostly due to overlooking the most significant aspects of an institutional arrangement in the health system.

**FIGURE 2:**
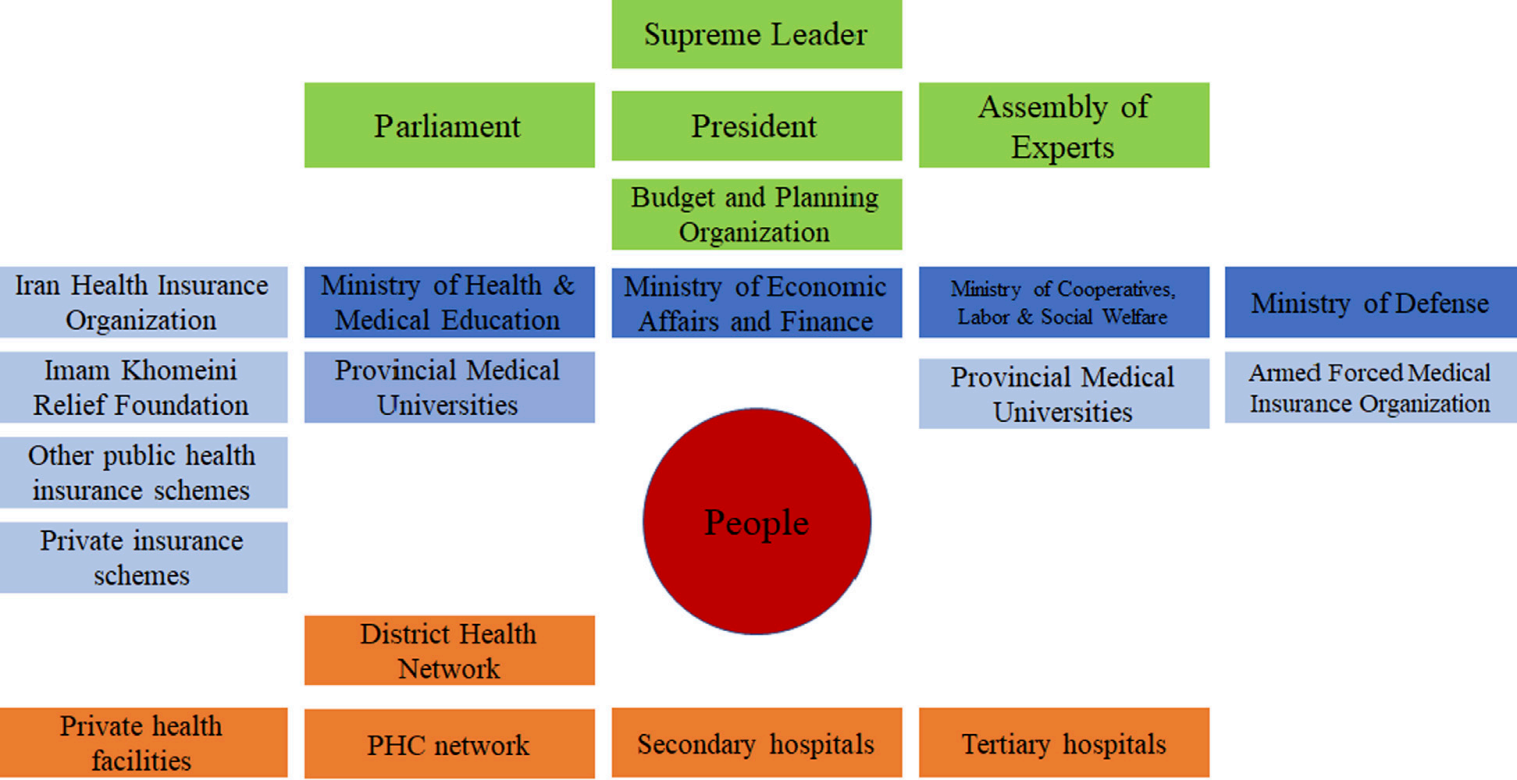
The health sector landscape in the Islamic Republic of Iran (Source from: World Health Organization, Geneva, 2020. License: Creative Commons Attribution CC BY-NC-SA 3.0 IGO) (Iran, 2020) [4].

Alignment of policymakers, academic groups, and civil society in tandem is one of the most important features of an institutional arrangement. Although the Iranian health system enjoys the existence of these three important groups at separate levels (Figure 2), fostering appropriate and effective relationships among them remains a concern [4]. For instance, the health system failed to utilize the capacity of medical universities for evidence-informed policymaking after the countrywide spread of the disease and faced some difficulties in forecasting proper estimations due to skewed use of the capacity of various scientific disciplines (e.g., health economics, policy, epidemiology, etc.) [9]. Besides, failure to engage the civil society in policymaking and insufficient transparency led to public distrustfulness and compromised compliance. As a result, people did not take some restricting policies seriously, therefore adherence to protocols deteriorated before the onset of each of the five COVID-19 waves in the country.

Another aspect of institutionalization that needs improvement is the role and function of the Iranian Center for Communicable Diseases Control (ICCDC) within the MoHME. The ICCDC is a relatively small department under the Health Deputy of the MoHME, which was assumed to play the role of a national center for disease control and prevention in Iran [13]. However, this consisted of multiple deficiencies, listed below:

• The weak legal framework to grant the ICCDC more authority, power, autonomy, and accountability to perform its functions and responsibilities [13];
• The meaningful deficiencies to collect and analyze timely and accurate data on COVID-19 cases, deaths, tests, and vaccinations, which hampered surveillance, monitoring, evaluation, and reporting activities [11];
• The lack of clear and consistent guidelines and protocols for COVID-19 diagnosis, treatment, isolation, quarantine, and contact tracing, for prevention and control of the disease [11];
• The inadequate number of qualified staff, equipment, supplies, and facilities to provide COVID-19 services, especially in rural and remote areas [11];
• The insufficient amount of effective coordination and collaboration with other departments and sectors of the MoHME, as well as with other stakeholders, such as provincial health authorities, medical universities, private providers, insurers, regulators, and civil society organizations [11];
• The lack of sufficient funding and financial support to implement COVID-19 interventions, especially in light of the economic sanctions and pressures [11].

Because of these factors, the ICCDC faced several challenges in fulfilling its role throughout various aspects of the COVID-19 response, such as testing, tracing, isolation, treatment, and vaccination, as well as in coping with the high burden of COVID-19 cases and deaths, and the emergence of new variants of the virus [13]. Therefore, it is essential to enhance institutionalization of the ICCDC within Iran’s health system to combat COVID-19 and other communicable diseases. This requires adopting a holistic and multi-sectoral approach that involves strengthening the capacity, governance, information system, coordination, collaboration, and funding of the ICCDC.

### Conclusion

The WHO declared that the COVID-19 outbreak can only be controlled by using all government capacities through a coherent and comprehensive approach, not just the capacity of the Ministries of Health. In hindsight, this may address the essential role of an institutional arrangement in preparing an organized system in order to take all stakeholders into consideration and build a resilient system to deal with the unpredictable global challenges. This might be achieved through access to sustainable resources and integrated decision-making, where various interests, political rivals, and civil society interplay appropriately. Indeed, countries that showed a better performance in combating COVID-19 put inter-sectoral collaboration, through a whole-of-government/whole-of-society approach for instance, at the heart of their institutional mechanism to deal with emergencies. Iran, similar to many other countries, needs to strengthen its institutional approach, aiming to improve its resiliency, along the pathway of preparation to face future likely pandemics, drawing upon lessons learned from the COVID-19 crisis.

## References

[1] Cho MJ Saravanan VS Kim EJ. Governing the Pandemics: Moving towards an Assertive Institutional Environment. J Glob Health (2021) 11:03021. 10.7189/jogh.11.03021 33643631 PMC7898558

[2] Zida A Lavis JN Sewankambo NK Kouyate B Moat K. The Factors Affecting the Institutionalisation of Two Policy Units in Burkina Faso’s Health System: a Case Study. Health Res Pol Syst (2017) 15(1):62. 10.1186/s12961-017-0228-2 PMC5512951 28716143

[3] Mayka L. The Origins of strong Institutional Design: Policy Reform and Participatory Institutions in Brazil's Health Sector. Comp Polit (2019) 51(2):275–94. 10.5129/001041519x15647434969830

[4] Hsu J Majdzadeh R Harichi I Soucat A. Health System Transformation in the Islamic Republic of Iran:an Assessment of Key Health Financing and Governance Issues. Geneva: World Health Organization (2017).

[5] Khanna R Cicinelli M Gilbert S Honavar S Murthy G. COVID-19 Pandemic: Lessons Learned and Future Directions. Indian J Ophthalmol (2020) 68(5):703–10. 10.4103/ijo.IJO_843_20 32317432 PMC7350475

[6] Raoofi A Takian A Haghighi H Rajizadeh A Rezaei Z Radmerikhi S COVID-19 and Comparative Health Policy Learning; the Experience of 10 Countries. Arch Iran Med (2021) 24(3):260–72. 10.34172/aim.2021.37 33878884

[7] Koon AD Windmeyer L Bigdeli M Charles J El Jardali F Uneke J A Scoping Review of the Uses and Institutionalisation of Knowledge for Health Policy in Low- and Middle-Income Countries. Health Res Pol Syst (2020) 18(1):7. 10.1186/s12961-019-0522-2 PMC6971874 31959208

[8] IMNA News Agency. Increasing Trend of "Omicron" in the Country (2022). Identification of 194 infected so far, Tehran2022 [updated 2022 Jan 6]. Available from: https://www.imna.ir/ . (Accessed January 6,2022).

[9] Gharebaghi R Heidary F. COVID-19 and Iran: Swimming with Hands Tied. Swiss Med weekly (2020) 150:w20242. 10.4414/smw.2020.20242 32255497

[10] Arab-Zozani M Ghoddoosi-Nejad D. COVID-19 in Iran: the Good, the Bad, and the Ugly Strategies for Preparedness - A Report from the Field. Disaster Med Public Health preparedness (2020) 15:e43–5. 10.1017/dmp.2020.261 PMC7588722 32713382

[11] Raoofi A Takian A Akbari Sari A Olyaeemanesh A Haghighi H Aarabi M. COVID-19 Pandemic and Comparative Health Policy Learning in Iran. Arch Iran Med (2020) 23(4):220–34. 10.34172/aim.2020.02 32271594

[12] Abdi M Coronavirus Disease 2019 (COVID-19) Outbreak in Iran: Actions and Problems. Infect Control Hosp Epidemiol (2020) 41:754–5. 10.1017/ice.2020.86 32192541 PMC7137533

[13] Moradi G Asadi H Gouya MM Nabavi M Norouzinejad A Karimi M The Communicable Diseases Surveillance System in Iran: Challenges and Opportunities. Arch Iran Med (2019) 22(7):361–8. 31679378

